# The APSANTICO Study: A Prospective Observational Study to Evaluate Antiphospholipid Antibody Profiles in Patients with Thromboembolic Antiphospholipid Syndrome (APS) after COVID-19 Infection and/or Vaccination

**DOI:** 10.3390/ijms24065644

**Published:** 2023-03-15

**Authors:** Olivia Ott, Eva Herrmann, Annabel Schulz, Edelgard Lindhoff-Last

**Affiliations:** 1 Coagulation Centre, Cardiology Angiology Centre Bethanien Hospital (CCB), 60389 Frankfurt, Germany; 2 Coagulation Research Centre Bethanien Hospital, 60389 Frankfurt, Germany; 3 Institute of Biostatistics and Mathematical Modelling, Goethe University, 60590 Frankfurt, Germany

**Keywords:** antiphospholipid syndrome, coronavirus disease 19, severe acute respiratory syndrome coronavirus 2, antiphospholipid antibodies, vaccination, thrombosis

## Abstract

Severe acute respiratory syndrome coronavirus 2 (SARS-CoV-2) is a new virus discovered in December 2019 that causes coronavirus disease 19 (COVID-19) and various vaccinations have been developed. The extent to which COVID-19 infections and/or COVID-19 vaccinations alter antiphospholipid antibodies (aPL) in patients with thromboembolic antiphospholipid syndrome (APS) remains unclear. Eighty-two patients with confirmed thromboembolic APS were included in this prospective non-interventional trial. Blood parameters including lupus anticoagulants, anticardiolipin IgG- and IgM-antibodies, and anti-ß2-glycoprotein I IgG- and IgM-antibodies were assessed prior to and after COVID-19 vaccination and/or COVID-19 infection. No increases in aPL in the total study population were detected. In fact, low but significant decreases were observed for anticardiolipin IgG- and anti-β2-glycoprotein I IgG-antibodies, while anticardiolipin IgM- and anti-b2-glycoprotein I IgM-antibodies slightly increased only in patients with COVID-19 infection and vaccination. Although the investigated patient group is known to have a high risk of recurrent thrombosis, only one arterial thrombotic event was diagnosed (1.2%, 1/82). This low recurrence rate was probably due to the high vaccination rates prior to infections and a high rate of effective anticoagulation. Our data show that COVID-19 infections and/or vaccinations do not deteriorate the clinical course of anticoagulated thromboembolic APS patients.

## 1. Introduction

Antiphospholipid syndrome (APS) is an autoimmune acquired thrombophilia with a prevalence of 1–5% in the general population [1]. It is characterized by the presence of antiphospholipid antibodies (aPL) as well as clinical symptoms. aPL are a heterogenous group of autoimmune antibodies directed against plasma proteins with high affinity to anionic phospholipids. 

The diagnosis of APS is made according to the revised Sydney Criteria [2], which are based on clinical and laboratory findings. On one hand, either lupus anticoagulants (LA), anticardiolipin IgM/IgG antibodies (aCL), and/or anti-β2-glycoprotein I IgG/IgM antibodies (anti-β2-GPI) must be persistently elevated on two occasions, a minimum of 12 weeks apart. On the other hand, venous and/or arterial thromboembolic events and/or pregnancy complications should have occurred.

APS can be divided into primary and secondary APS. While secondary APS is associated with autoimmune diseases such as systemic lupus erythematodes (SLE), primary APS cannot be attributed to an underlying autoimmune disease.

Due to the etiology of APS, many concerns about the management of patients with APS arose with the onset of the severe acute respiratory syndrome coronavirus 2 (SARS-CoV-2) pandemic.

SARS-CoV-2 is a new virus that emerged in December 2019 and causes the coronavirus disease 19 (COVID-19), with new waves of infections ever since. The current literature suggests that patients who have been or are infected with COVID-19 are at higher risk for thrombosis. This increased risk may be due to damaged endothelium, observed hypercoagulability, neutrophil extracellular traps, and macro- and microthrombosis [3]. 

Only less than a year after the discovery of SARS-CoV-2, the first mRNA vaccine was licensed in Germany, followed by further mRNA-based, adenovirus-vector-based vaccines and other vaccines [4]. 

Since APS and COVID-19 infections are diseases that are associated with a high risk of thrombosis, the impact of COVID-19 infections and/or vaccinations on patients with APS is of major interest [3]. It has been described that COVID-19 patients may have a higher prevalence of transiently occurring lupus anticoagulants [5]. The extent to which COVID-19 infection and/or vaccinations may affect the antibody profiles of patients with thromboembolic APS is unknown thus far and is being evaluated in the prospective APSANTICO study.

## 2. Results

### 2.1. Patient Characteristics

At the baseline, patients were on average 52 years old with a slightly higher proportion of women (53.7%, see Table 1 for more details). The majority of patients presented with cardiovascular risk factors (74.4%, 61/82), predominantly hypertension (53.7%) and hypercholesterolemia (48.8%). Sixty-five patients (79.3%) had developed venous thromboembolism and 32 patients (39%) presented with arterial thromboembolic events in the past. 

A total of 76.8% (63/82) of patients suffered from primary and 23.2% (19/82) from secondary APS including 11 patients with SLE. At the baseline, 32 patients were “triple-positive” (39%), 19 were “double-positive” (23.2%), and 31 “single-positive” (37.8%) (see Table 1). 

Autoimmune disease was present in 29 (35.4%) of the patients, with predominantly more women affected (69%, 20/29). In total, 20.7% (17/82) of the patients received immunosuppressive drugs.

All patients except one were anticoagulated (98.8%, 81/82) at the baseline. Most patients were treated with either vitamin K antagonists (VKA) (34.1%, 28/82) or direct oral anticoagulants (DOAC) (32.9%, 27/82). The remaining patients were anticoagulated with low molecular weight heparin (LMWH), fondaparinux, platelet inhibitors (PI), or combinations of coagulation inhibitors (see Table 2).

At follow-up, the majority of patients (91.5%, 75/82) had been vaccinated against COVID-19 at least once (see Table 3). Less than half had been infected with SARS-CoV-2 (40.2%, 33/82), of whom nearly one third had been taking immunosuppressive drugs at the time of infection (27.3%, 9/33). 

Most SARS-CoV-2 infections were observed in 2022 (74.3%, 26/35), whereas 22.9% (8/35) and 2.9% (1/35) were reported in 2021 and 2020. 

During the course of the study, no cases of venous thrombosis occurred, and only one patient experienced an arterial cerebral thromboembolism. Furthermore, we observed one case of cerebral bleeding (for more details, see Section 2.3).

### 2.2. Antiphospholipid-Antibodies and Platelet Counts at Baseline and at Follow-Up (after COVID-19 Infection and/or Vaccination)

In general, there were only a few changes in the aPL levels after COVID-19 vaccination and/or infection. 

At the baseline, 58.5% (48/82), 80.5% (66/82), 69.5% (57/82), and 34.2% (28/82) of the patients had lupus anticoagulants, elevated aCL IgG/IgM-antibodies, elevated anti-β2-GPI IgG/IgM-antibodies, and elevated anti-β2-GPI domain I IgG-antibodies (anti-β2-GPI domain I), respectively (see Table 1). 

No clinically relevant changes of the aPL-profiles were observed at follow-up (lupus anticoagulants: 61%, 50/82; aCL IgG/IgM-antibodies: 81.7%, 67/82; anti-ß2-GPI IgG/IgM-antibodies: 63.4%, 52/82; anti-ß2-GPI domain I: 37.8%, 31/82) (see Table 3). 

In addition, single-, double-, and triple-aPL positivities remained quite stable throughout the study. We observed an increase in aPL positivity in 7.3% of patients, a decrease in aPL positivity in 14.6% and a loss of aPL in 4.9% of patients, respectively. Of the four patients with a loss of their aPL, three had been vaccinated and one had been vaccinated and infected. 

**Figure 1:**
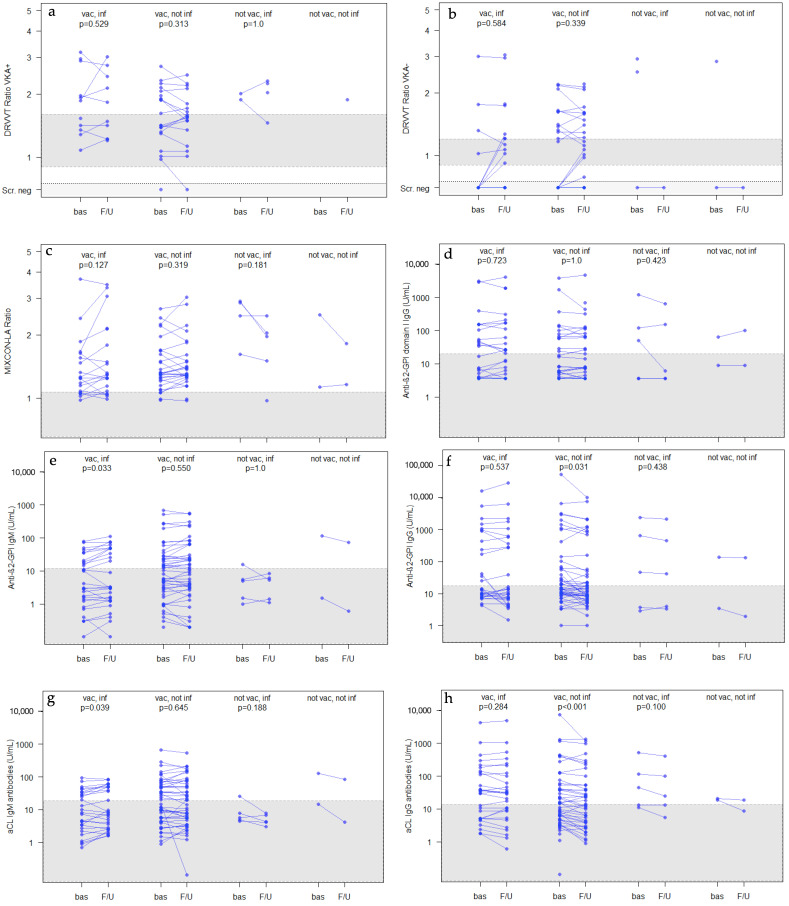

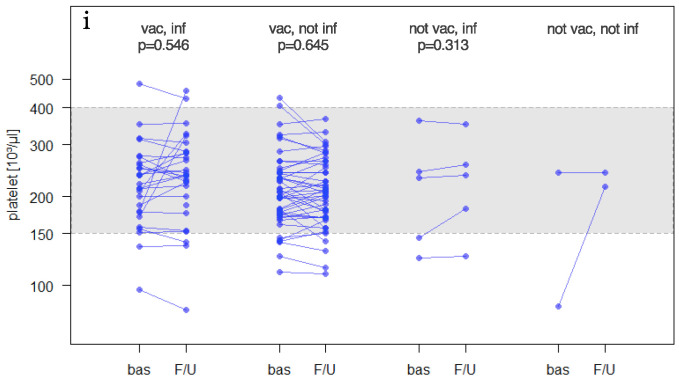
Course of different aPLs and platelet counts for different subgroups according to vaccination and infection status. Panels (**a**–**c**) show lupus anticoagulants (DRVVT ratio and screening with (+) and without (−) VKA and MIXCON LA ratio), panel (**d**) shows anti-β2-GPI domain I IgG antibodies, panels (**e**,**f**) show anti-β2-GPI IgM- and IgG-antibodies, panels (**g**,**h**) show aCL IgM- and IgG-antibodies, and panel (**i**) shows the platelet counts. The vertical axes are on log-scale. *p*-values were calculated with the Wilcoxon-matched pairs test on a logarithmic scale. The shaded area shows the reference ranges of each assay. Points are shaded and a stronger color is caused by overlaying the data points. The DRVVT ratio is only evaluated in patients with positive DRVVT screening. Only these quantifications were statistically compared but the respective panel also illustrates cases with negative screening (with overlaying data points). vac.: COVID-19 vaccination; inf.: COVID-19 infection; Scr. neg: screening negative; bas: baseline; F/U: follow-up; DRVVT: dilute Russell’s viper venom time; VKA+: patients on vitamin K antagonist treatment; VKA–: patients without vitamin K antagonist treatment; MIXCON-LA: a lupus-sensitive aPTT-based assay; Anti-β2-GPI: Anti-β2-Glycoprotein I; IgG: Immunoglobulin G; IgM: Immunoglobulin M; aCL: anticardiolipin antibody; platelet: platelet count.

The dilute Russell’s viper venom time (DRVVT) ratio was measured separately for patients with and without VKA intake. For both patient groups, there were no significant changes from the baseline to follow-up in the total study group and in all sub-groups (see Figure 1a,b and Table 4). In addition, no significant changes were observed for lupus anticoagulants measured by the MIXCON LA ratios (an aPTT-based lupus anticoagulant assay) at follow-up (see Figure 1c and Table 4). 

In contrast, anti-β2-GPI IgG-antibodies decreased in all study groups with minor but significant reductions in the total study population (*p* = 0.021) and in patients with COVID-19 vaccination only (*p* = 0.031, see Figure 1f and Table 4). However, anti-β2-GPI IgM significantly increased in patients with COVID-19 vaccinations and infection, even so, the empirical medians decreased (*p* = 0.033, see Figure 1e and Table 4).

During the observation period, there were no significant changes in anti-β2-GPI domain I antibodies (see Figure 1d and Table 4).

There was also a significant decrease in the aCL IgG-antibody levels in the total study population and in the patients with COVID-19 vaccination only (*p* < 0.001, see Figure 1h and Table 4). For aCL IgM antibodies, slight decreases in the empirical medians were observed in all groups. However, in patients with COVID-19 vaccination and infection, a slight but significant increase was observed with the Wilcoxon matched-pairs test (*p* = 0.039, see Figure 1g and Table 4).

Platelet counts (see Figure 1i and Table 4), antinuclear antibodies (ANA), and anti-double stranded deoxyribonucleic acid antibodies (anti-dsDNA) remained unchanged during follow-up.

### 2.3. Hospital Admissions Due to COVID-19 Infection or COVID-19 Vaccination

#### 2.3.1. Hospital Admissions Due to COVID-19 Infection (see Table 5)

During the course of the study, three patients were admitted to hospital due to COVID-19 infection (3.7%, 3/82). The first patient, a 77-year-old female, was admitted to hospital 11 days after a positive COVID-19-PCR with minor symptoms (nausea, vomiting, headache). She was single-positive for aPL. Prior to the positive COVID-19 testing, the patient had received three COVID-19 vaccinations (mRNA vaccine Comirnaty^®^ by BioNTech/Pfizer, first and second vaccination 21 days apart, second and third vaccination about 9 months apart). She was discharged from hospital 5 days after admission and did not show persistent symptoms. 

The second patient was a 38-year-old female, with triple-positive APS and SLE who had been vaccinated twice (Comirnaty^®^ vaccine by BioNTech/Pfizer, vaccination dates 28 days apart). She was infected with COVID-19 (positive PCR test) about 4.5 months after the second vaccination. Eleven days after the start of her infection, she was admitted to hospital for one day due to esophagitis. 

The third patient admitted to hospital because of a COVID-19 infection (positive PCR test) was a 60-year-old woman naïve to COVID-19 vaccinations. She was single-positive for aPL and had an underlying myeloproliferative disorder with a positive Janus kinase 2 gene mutation. On admission, she presented with symptoms of acute general distress. The patient’s condition increasingly deteriorated, leading to her being transferred to the intensive care unit and requiring ventilation for more than a month due to respiratory insufficiency. Overall, the patient was discharged from the hospital 7 weeks after admission in a stable but impaired general condition. 

#### 2.3.2. Hospital Admissions Due to COVID-19 Vaccination (see Table 5)

In total, four patients (4.9%, 4/82) were hospitalized after COVID-19 vaccination. 

The first case was a 45-year-old woman with a history of rheumatoid arthritis and single-positive APS. She developed adhesive capsulitis seven days after her second vaccination (Comirnaty^®^ vaccine, BioNTech/Pfizer). Her ambulant treatment included high-dose cortisone. The patient was admitted to hospital 37 days later with persistent spinning vertigo, with periods of aphasia and vomiting. A MRI of the brain was conducted, which ruled out ischemia and sinus thrombosis. The symptoms declined, a benign paroxysmal positional vertigo was suspected, and she was discharged 3 days after admission. 

The second case was a 71-year-old male APS patient with double-positive aPL, who had received three vaccinations with Comirnaty^®^ (BioNTech/Pfizer). He was admitted to the hospital two days after the third vaccination due to an acute left-sided hearing loss, which was treated with cortisone. The patient’s hearing was 75% restored 3 weeks after the event. 

The third patient, an 85-year-old single-positive male, was admitted to hospital five days after his second vaccination with Comirnaty^®^ (BioNTech/Pfizer). He presented with moderate ataxic left-sided arm paresis and dysarthria. A paramedian pons infarction, most likely of microangiopathic genesis, was diagnosed and the patient left the hospital 19 days after admission. Medication with apixaban was continued as prior to the event and the third COVID-19 vaccination 8.5 months after the event was tolerated well without any complications. This was the only case of thrombosis during the observation period in the whole study population.

The fourth case was a 54-year-old male. He had a triple-positive APS and was anticoagulated with warfarin (target international normalized ratio, INR, 3–4) and acetylsalicylic acid (ASS) 100 mg twice daily. This intensive dual therapy was necessary due to repeated venous thrombosis despite anticoagulation with VKA and due to aPL interfering with the INR-measurements, leading to falsely high INR-levels in the past. Thirty-three days after his second COVID-19 vaccination with Comirnaty^®^ (BioNTech/Pfizer), he developed dysesthesia of the right hand, and a spontaneous subacute subdural hematoma was diagnosed after admission. At that time, the INR was 5.0 despite unchanged VKA-medication. Warfarin and ASS were paused, but after the patient had developed aphasia, a surgical intervention with the removal of the subdural hematoma became necessary. During his stay in hospital, the patient received four transfusions of fresh frozen plasma and two platelet concentrate transfusions. Immediately after the event, the patient was switched from warfarin to enoxaparin, then to fondaparinux, and is currently anticoagulated with apixaban and ASS. A vaccine-associated coagulopathy was suspected due to the detection of platelet-factor 4 IgG antibodies and a positive platelet factor 4 enhanced heparin-induced platelet activation (PIPA) test, which occurred after the spontaneous intracranial bleeding.

### 2.4. INR Variations Due to COVID-19 Infection or COVID-19 Vaccination

In addition to the patient described above, three other VKA-treated patients showed INR variations either after COVID-19 infection or after COVID-19 vaccination (11.8%, 4/34). 

The first patient was a 59-year-old female with a triple-positive APS. She was infected with COVID-19 but had a mild course of the disease. Without changing the dose of phenprocoumon (target INR 3–4), the INR value spontaneously rose to values between 5.0 and 5.8 for two weeks. There was no increased bleeding tendency during this period. Within four weeks, the INR returned to the target INR. 

The second case was a 47-year-old female with a single-positive secondary APS associated with SLE. She received warfarin with a target INR of 2–3. Shortly after her second vaccination with Comirnaty^®^ (BioNTech/Pfizer), she noticed that the INR-values spontaneously increased to 5.9 and 8.0, without any bleeding complications. In reaction to these high INR-values, warfarin was terminated and a switch to apixaban was made. When the INR and a chromogenic factor X assay were measured at the time of the spontaneous INR-increase, it became evident that the self-monitored INR assessed by the patient was measured as falsely high due to interfering antiphospholipid antibodies. This interference had not occurred before the COVID-19 vaccinations. Additionally, the blood values showed newly developed lupus anticoagulants as well as positive antibodies against platelet-factor 4. Therefore, it was assumed that she suffered from a rare vaccine-associated coagulopathy with positive platelet factor 4 induced platelet activating (PIPA)-antibodies potentially induced by the second COVID-19 vaccination.

In contrast, the third patient repeatedly developed a decrease in his INR after his COVID-19 vaccinations and infection. He was a 51-year-old male with a secondary triple-positive APS receiving phenprocoumon with a target INR of 2.0–3.0. After COVID-19 infection, the INR decreased to 1.5, necessitating a dose increase of phenprocoumon. In addition, the INR levels decreased after both vaccinations with Comirnaty^®^ (BioNTech/Pfizer). For this reason, a booster vaccination was not recommended.

## 3. Discussion

Patients with thromboembolic APS are at a high vascular risk for both thromboembolic complications because of the disease and bleeding events because of medication with anticoagulants and/or platelet inhibitors, which is mandatory in almost all cases. In early 2020, the worldwide COVID-19 pandemic started, with many people infected. Since APS and COVID-19 infections are diseases that are associated with a high risk of thrombosis, the impact of COVID-19 infections and/or vaccinations on the aPL-profiles of patients with thromboembolic APS was investigated in the APSANTICO-study.

### 3.1. aPL and COVID-19 Vaccination/Infection

It is known that aPL can appear temporarily in severe illnesses and various infections other than COVID-19 [6]. Since the beginning of the COVID-19 pandemic, many publications have therefore analyzed the occurrence of aPL in a case series of COVID-19 infected patients [5,7,8,9,10,11].

Gendron et al. examined aPL in 154 hospitalized patients with COVID-19 infection compared to 95 hospitalized non-COVID-19 patients. Although the incidence of LA positivity was significantly higher in the COVID-19 patients (60.9% vs. 23.7%, *p* < 0.001), LA positivity was not associated with an increased venous thromboembolism risk or mortality [5].

Another study analyzing 24 hospitalized patients suffering from severe COVID-19 infection also evaluated the presence of aPL during infection. All patients had COVID-19 pneumonia and venous thromboembolism and did not have previously diagnosed APS. Only two showed weakly positive aCL IgM- and anti-β2-GPI IgG-antibodies and three developed thrombocytopenia. Thus, the authors suggested a low prevalence of new onset aPL in patients with severe COVID-19 infections [8]. 

Additionally, Borghi et al. reported that only a few patients with severe COVID-19 infection showed aPL, and the titers were only medium to low in contrast to patients with APS, who displayed rather high titers. Additionally, the authors mentioned that aPL differed in domain specificity in patients with COVID-19 compared to patients with APS. Furthermore, they also stated that aPL in COVID-19 patients did not increase the risk of thrombosis [9]. 

This agrees with another study of 158 hospitalized patients with COVID-19 infection in whom 16 patients were persistently positive for aPL, but only two of them suffered from thrombosis during hospitalization. Most determinations were at low titers, and they were not related to worse clinical outcomes [10].

In contrast, Zuo et al. examined the aPL IgG fractions isolated from 172 patients hospitalized with COVID-19 infection and observed that these fractions promoted neutrophil extracellular trap release from neutrophils isolated from healthy individuals. Furthermore, these IgG fractions accelerated venous thrombosis in two mouse models. Half of the COVID-19 infected patients became at least transiently positive for aPL antibodies, and the authors suspected that these autoantibodies were potentially pathogenic [11].

In contrast to the studies above-mentioned, our patients were all positive for aPL prior to infection with SARS-CoV-2, and only very few needed hospitalization due to COVID-19 infection. Unexpectedly the aPL in our total study group did not rise any further after the COVID-19 infection and/or vaccination. In fact, we observed a significant though minor reduction in aCL IgG antibodies and anti-β2-GPI IgG antibodies in APS patients with COVID-19 infection with or without vaccination. However, in patients who were both vaccinated against and infected with COVID-19, we observed low but significant increases in the aCL IgM antibodies and anti-b2-GPI IgM antibodies, respectively. The clinical relevance of our findings is still unclear. All in all, the only slight antibody fluctuations indicate a rather low impact of COVID-19 vaccinations and/or infections on aPL in patients with thromboembolic APS.

### 3.2. Thrombocytopenia and COVID-19 Vaccination/Infection

Thrombocytopenia has been described to be associated with COVID-19 vaccination with both vector-based and mRNA-based COVID-19 vaccines as well as with COVID-19 infections [12,13]. Thrombocytopenia is also the most common non-criteria hematological feature in patients with APS [14].

A recent single-centre German study reported a 2-fold increase in immune thrombocytopenia (ITP) cases since COVID-19 vaccinations were introduced at the end of 2020 [15]. The authors described nine out of 31 patients with COVID-19 vaccination related ITP with a median platelet count of 5/nL at the time of admission. Furthermore, two patients were diagnosed with ITP associated with COVID-19 infection.

It is known that various infectious diseases can cause thrombocytopenia such as hepatitis C virus, human immunodeficiency virus, Helicobacter pylori, SARS-CoV, and SARS-CoV-2 [16,17,18]. 

In our study, we assessed the platelet count in all patients before and after COVID-19 vaccination/infection. No significant changes were observed, even though 40.2% of our patients were infected with COVID-19 at least once (Table 3).

Pontara et al. evaluated platelet counts of 119 consecutive high-risk triple-positive APS patients over time independently from infections [14]. Patients were divided into sub-groups of APS without associated autoimmune disease (PAPS), patients with associated autoimmune disease (SAPS), and patients who developed a catastrophic APS (CAPS). For patients with PAPS and patients with SAPS, prevalence of thrombocytopenia (cut-off 100 10^3^/µL) was low (6%) and the two groups did not show a remarkable difference in platelet counts (7% PAPS and 3% SAPS). In contrast, 100% of CAPS patients showed thrombocytopenia on the day of the CAPS diagnosis. The authors concluded that the prevalence of thrombocytopenia was low, even in this high risk APS population and that a decrease in platelet count should be considered as a warning signal for disease progression to CAPS.

Our study population consisted of single-, double-, and triple-positive APS patients, who also did not develop new thrombocytopenia, although most were infected with and/or vaccinated against COVID-19. Interestingly none of our patients developed CAPS.

### 3.3. The Immune System and COVID-19 Vaccination/Infection

It is suspected that COVID-19 infections and COVID-19 vaccinations may cause immune dysregulation, leading to increased occurrences of autoimmune diseases after infection and vaccination [19,20].

A prospective study of 33 patients with COVID-19 pneumonia analyzed ANA, aPL, and anti-neutrophil cytoplasmic antibodies (ANCA). The authors reported that patients who were positive for at least one autoantibody class had a more severe course of the disease than patients without autoantibodies, suggesting an induced immune dysregulation due to COVID-19 [21]. 

In 2005, Gómez-Puerta et al. published a long-term follow-up study on 128 patients with primary APS, with the specific question of whether they developed SLE or not [22]. After a median disease duration of 8.2 years (range, 1–14 y), 86% patients still had primary APS and only 8% had developed SLE, 5% lupus-like disease (LLD), and 1% myasthenia gravis, respectively. In summary, they estimated a low risk of progression from primary APS to SLE or LLD, with the limitation that this transition may take even longer than the trials’ follow-up. 

When we started our investigations, we suspected that COVID-19 infections or vaccinations could trigger the immune system of APS patients, which may lead to an increased conversion from primary APS to secondary APS thereafter. Considering that our follow-up was conducted after a short period of time, it may not come as a surprise that we did not find any significant changes in the median ANA and anti-dsDNA antibody titers after COVID-19 infections and/or vaccinations, although one might have assumed that COVID-19 infections and /or vaccinations could accelerate the transition from primary to secondary APS. 

### 3.4. COVID-19 Infections and COVID-19 Vaccinations and Their Association with Thromboembolic Complications

Contrary to our expectations, we found that none of our patients experienced thrombosis after COVID-19 infection, although this patient population is known to have a high thrombosis-recurrence risk. 

Only one patient developed an arterial thrombosis after his second COVID-19 vaccination with a mRNA vaccine. 

Previous studies have shown that an infection with SARS-CoV-2 is accompanied with a higher risk for thrombosis. For example, at the beginning of the COVID-19 pandemic, Zou et al. reported significantly higher coagulation parameters in patients with severe COVID-19 infections compared with those having a mild course of the disease. Still, both groups had elevated coagulation parameters, resulting in a higher risk for thrombosis during the infection [23].

A possible reason for our finding is the fact that the majority of our patients (78.8%) had been vaccinated before they developed a COVID-19 infection. 

A review published in February 2022 described an mRNA vaccine effectiveness against hospitalization or death of 89–95% against the SARS-CoV-2 Alpha variant, 95% against Beta/Gamma, 80–95% against Delta, and 87–95% when the virus strain was not sequenced, resulting in a high level of protection against severe COVID-19 infection and hospitalization for all variants after immunization [24]. In addition, mRNA vaccination protection against hospitalization due to Omicron infection is reported to be 90.9% [25].

Furthermore, the majority of our patients experienced COVID-19 in 2022 (74.3%, 26/35), whereas only 22.9% and 2.9% were infected in 2021 and 2020, respectively. The strain dominant in Germany in 2022 was Omicron, being responsible for more than 95% of all SARS-CoV-2 infections, while Delta led in the second half of 2021 (up to 99% of all infections) and Alpha was predominant in the first half of 2021 (about 90% of all infections) [26]. 

It is known that infections with the Omicron variant cause less severe courses of disease and hospitalization than the other variants [27]. 

As discussed earlier, the risk for thrombosis in mild courses of the disease was lower, hinting that most of our patients were at lower risk of thrombosis due to the variant they were probably infected with and their vaccination status prior to the infection. 

Another possible reason for the low number of recurrent thrombosis in our study population is the protection provided by anticoagulants in nearly all of our patients.

In contrast, COVID-19 vaccinations might be associated with rare complications. Greinacher et al. described a case series of eleven patients suffering from mild to severe thrombocytopenia and unusual thrombosis 1–2 weeks after vaccination with the adenovirus-based vaccine by AstraZeneca. Symptoms were similar to those in heparin-induced thrombocytopenia (HIT), although none of the patients received heparin prior to the events. The patients’ sera were also strongly positive for platelet factor 4 (PF4) antibodies. The authors described this finding as vaccine-induced immune thrombotic thrombocytopenia (VITT) [28]. While this rare complication was mainly associated with adenovirus-based vaccines, a recently published case report described a 66-year-old woman who suffered from deep leg vein thrombosis three days after vaccination with an mRNA vaccine (Comirnaty^®^; BioNTech/Pfizer) [29]. These vaccination complications remain very rare but still need to be kept in mind. 

In our study, only one patient developed an arterial thrombosis despite VKA-treatment 5 days after his second mRNA vaccination with Comirnaty^®^ (BioNTech/Pfizer). 

### 3.5. Strengths and Limitations

#### 3.5.1. Strengths of the Study

We have been treating patients with APS at our outpatient clinic for many years. Annual check-ups including blood sampling to determine aPL are carried out regularly. In addition, we preserve the residual citrated plasma at every outpatient visit for possible later examinations (biobank). Thus, we have one of the largest APS patient populations in Germany, from which data were available before the onset of the COVID-19 pandemic. Most of our patients were willing to participate in studies, so we were able to include many patients with thromboembolic APS (i.e., a patient group with a high risk of recurrent thrombotic events (n = 82)). After COVID-19 vaccines were introduced at the end of December 2020, more than 90% of our patients (n = 75) were vaccinated, mostly with the mRNA vaccine alone (87%, n = 65). Therefore, we were able to generate a lot of data from thromboembolic APS patients who had previously been vaccinated and could compare these results to their baseline values. Furthermore, during the very high daily COVID-19 new infections in spring 2022 in Germany, 31.7% (26/82) of our patients additionally became infected with SARS-CoV 2 despite previous vaccination (88.5%, 23/26), giving us the opportunity to also collect data in this patient group.

#### 3.5.2. Limitations of the Study

Due to the non-interventional nature of our study, we were dependent on when the patients were able to attend their next check-up appointment. Thus, the temporal intervals of blood sampling in the follow-up period varied in relation to the events (e.g., the interval between the follow-up blood sampling and the last COVID-19 disease or vaccination). Baseline parameters were taken from the last examination before the first COVID-19 vaccination or infection, whichever came first. Therefore, interindividual variations in the time intervals between the blood collection time and the event also occurred here. Furthermore, only the data of two blood collections per patient could be compared (baseline versus follow-up), so we could not make any statements about the time courses of the aPL levels directly after a COVID-19 infection or vaccination. We did not have any influence on the vaccination rate of our patients. Furthermore, we could not control the number of patients infected with COVID-19 during the pandemic. High-risk patient groups were advised to get vaccinated to avoid possible thromboembolic events caused by the potentially high thrombogenic COVID-19 infection. Accordingly, most of our patients had been vaccinated and also partially infected, resulting in a very small number of patients only infected (n = 5) or patients with neither a COVID-19 vaccination nor infection (n = 2). This led to a heterogenous distribution of patients in the subgroups.

## 4. Materials and Methods

The population of the prospective APSANTICO observational study was generated based on the patient population of the APSantiCO registry previously published [30]. In brief, this was a patient group with thromboembolic APS who matched the Sydney classification criteria for APS [2]:

- Minimum age of 18 years;
- Presence of any APS antibody risk profile (single-/double-/triple-positivity);
- Arterial and/or venous thromboembolism in their medical history.

Patients with sole obstetric APS were excluded from the study.

After receiving the positive ethics vote on 15 March 2022, all 80 patients of the APSantiCO registry were screened for existing APS antibody titers before a COVID-19 infection and/or vaccination. The majority of APS patients treated at the Coagulation Centre at Bethanien Hospital, Frankfurt, Germany are routinely examined once every 6 to 12 months to monitor aPL-titers and reevaluate their current anticoagulant medication. Patients of the APSantiCO registry who had already scheduled a routine appointment in the outpatient clinic within the next 6 months were informed of the study and screened during their routine visit (n = 24). Patients who had not yet scheduled their next routine appointment (n = 56) were informed about the study by an information letter and a telephone call. If the patient was interested in participating in the study, an appointment for the next check-up was scheduled. Patients who wanted to participate in the study gave their written informed consent and were included. At that visit, blood sampling and a routine medical examination were performed.

**Figure 2:**
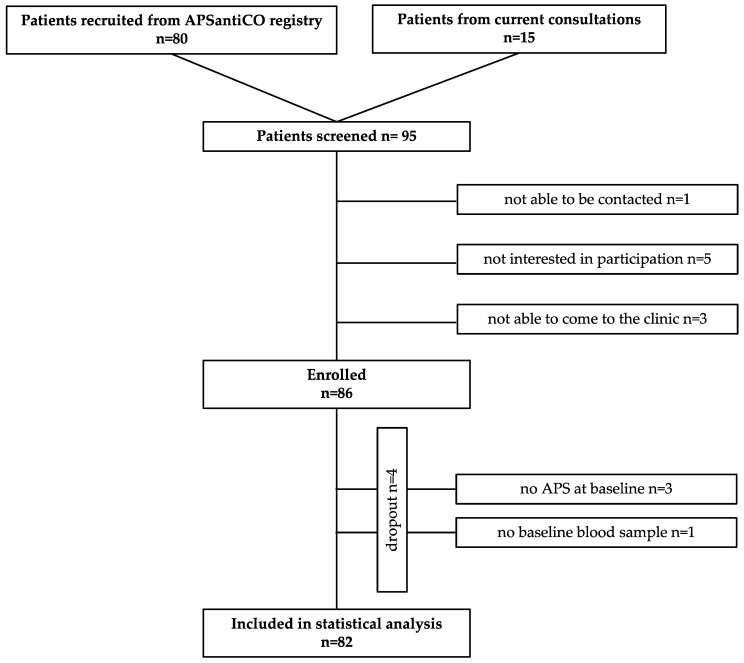
Flowchart of patient inclusion.

In addition to the 80 patients screened as part of the APSantiCO registry, 15 patients were screened from the current consultations, resulting in 95 screened patients in total. Of these, 86 patients were enrolled in the study. Four patients were screening failures who either did not fulfil the criteria of thromboembolic APS at the baseline (n = 3) or had not been in the outpatient clinic before the first COVID-19 vaccination (n = 1). A total of 82 patients gave their written informed consent and were included in the final statistical analysis (see Figure 2).

**Figure 3:**
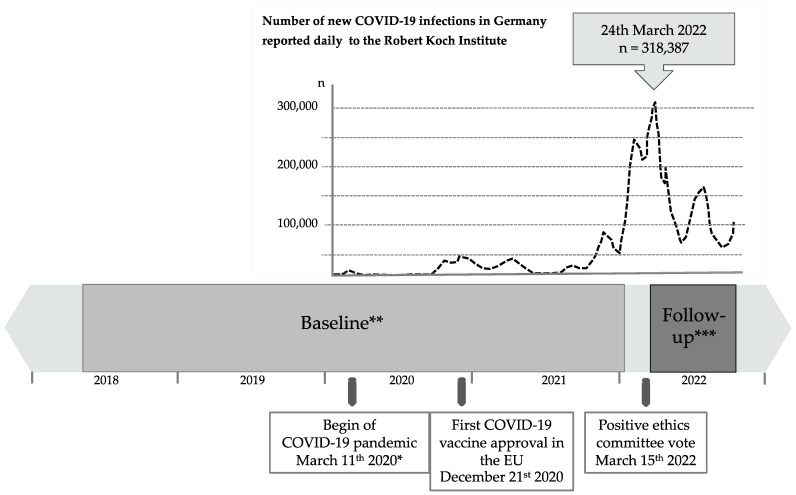
Timeline of blood sampling. The figure shows the number of new COVID-19 infections in Germany based on data provided by the Robert Koch Institute [31]. * The global spread of COVID-19 was declared a pandemic by the WHO on 11 March 2020. ** Baseline blood parameters were taken from each patient’s medical record. All blood specimens were taken before the first COVID-19 infection or COVID-19 vaccination (median: 200 days; 25–75% percentiles: 102.5–354.0; timeframe: 2 May 2018–6 January 2022). *** Follow-up blood parameters were taken after the last COVID-19 infection or COVID-19 vaccination at the patient’s visit to the outpatient clinic (median: 142 days; 25–75% percentiles: 88.5–199.0; timeframe: 17 March 2022–24 October 2022). Two patients were neither infected nor had they been vaccinated. In these patients, the time span between the baseline and follow-up blood parameters was 434 and 177 days, respectively.

Clinical data such as gender, age, comorbidities, cardiovascular risk factors, medical history, and medication were retrieved from each patient’s electronic medical record. For the patients’ baseline blood parameters, we used the values that were analyzed before their first COVID-19 infection or COVID-19 vaccination. The follow-up values were analyzed in the blood samples taken after we had received a positive ethics committee approval (15 March 2022, see Figure 3). Fortunately, the follow-up period of our study fell at a time just after the COVID-19 pandemic peak, so almost all our patients had either been vaccinated against and/or had been infected with SARS-CoV-2. 

The following blood parameters were assessed: platelet count, c-reactive protein (CRP), antinuclear antibodies (ANA), anti-double strand deoxyribonucleic acid (dsDNA), and aPL.

At their outpatient visit, patients were asked about their COVID-19 vaccinations and/or COVID-19 infections. Furthermore, the side effects of each vaccination and any complications during/after COVID-19 infection were documented. Complications such as thrombosis, embolism, or bleeding associated with vaccination and/or infection were assessed. Major bleeding (MB) and clinically relevant non-major bleeding (CRNMB) were classified as per the ISTH criteria [32].

Whether the patient suffered from secondary rather than primary APS was decided by the presence of autoimmune diseases such as SLE, Sharp syndrome, and Sjögren’s syndrome, as found in the patient’s electronic medical record. Additionally, anticoagulant medication was documented. 

To measure the titers of anti-β2 glycoprotein I IgG- and IgM-, anticardiolipin IgG- and IgM-antibodies and antibodies against domain I of the ß2-glycoprotein IgG, a fully automated chemiluminescence immunoassay was used (Hemosil^®^ AcuStar Anti-Cardiolipin IgG and IgM; Hemosil^®^ AcuStar Anti-β2-Glycoprotein-I IgG and IgM, Hemosil^®^ AcuStar Anti-ß2-Glycoprotein-I Domain I, Instrumentation Laboratory, Bedford, MA, USA). 

To measure the parameters of lupus anticoagulants, dilute Russell’s viper venom time assays (dRVVT, Hemosil^®^ dRVVT Screen and Hemosil^®^ dRVVT Confirm, Instrumentation Laboratory, Bedford, MA, USA) and a lupus-sensitive aPTT-based assay (MIXCON LA-test, Instrumentation Laboratory, Kirchheim, Bavaria, Germany) were used. It is known that the intake of DOAC can alter the DRVVT ratio. Therefore, we added DOAC Remove^TM^ (5–Diagnostics AG, Basel, Switzerland) to the blood samples prior to performing the DRVVT testing in the DOAC treated patients. Furthermore, the DRVVT ratio was also influenced by the VKA intake. For this reason, the reference ranges differed between the patients with and without VKA treatment. 

Measurement of ANA was performed using fluorescence microscopy and anti-dsDNA analysis was performed with a fluorescent enzyme immunoassay (Phadia^TM^ 250 by Thermo Fisher Scientific, Waltham, MA, USA).

The study was registered at ClinicalTrials.gov: NCT05313048 and ethics approval was obtained from the local Ethics Committee (approval number 2021-2716-evBO).

### Statistical Analysis

The main observational outcome measure was the change in APS antibody titers and platelet counts after COVID-19 vaccination and/or infection, subdivided into patients who only received COVID-19 vaccination, who received COVID-19 vaccination, and were infected with SARS-CoV-2, and those who were only infected with SARS-CoV-2. Furthermore, we investigated possible changes in APS positivity and changes in ANA and anti-dsDNA antibodies. 

Within group comparisons for the qualitative and quantitative data of the total study population and subgroups were performed using Wilcoxon matched-pairs test on a log-scale and McNemar test. No tests were performed in subgroups with only two patients. A log scale (y-axis) was also used in the figures. Data were summarized by counts (percentages) and the median with range (25–75% percentile).

For statistical analysis, JASP Team (2022, Version 0.16.4 Intel), University of Amsterdam, the Netherlands on macOS Monterey (Version 12.2.1) and R (R Foundation for Statistical Computation, Vienna, Austria, Version 4.1.0) were used.

## 5. Conclusions

In summary, neither COVID-19 infection nor vaccination or the combination of both showed clinically relevant deleterious effects on the aPL titers of patients with thromboembolic APS. We observed some minor but significant decreases in aCL IgG and anti-β2-GPI IgG after COVID-19 infection and/or vaccination and some minor but significant increases in aCL IgM and anti-b2-GPI IgM in patients who were both vaccinated against and infected with COVID-19. Four patients lost their positivity regarding aPL at follow-up. Although our total study group is known to have a high risk of thrombosis recurrence, we only observed one arterial thrombotic event (ATE) in a male, 85-year-old patient during the COVID-19 pandemic. The low incidence of thrombotic events in our study is probably due to a high rate of vaccinations prior to COVID-19 infection and an effective, continuous anticoagulant medication in the majority of patients at the baseline and during follow-up. 

In conclusion, our data show that COVID-19 infections and/or vaccinations do not deteriorate the clinical course of anticoagulated thromboembolic APS patients.

## Figures and Tables

**Table 1:** Patient characteristics at the baseline.

	Total Study Population (n = 82)	COVID-19 vac. only (n = 47/82)	COVID-19 vac. and inf. * (n = 28/82)	COVID-19 inf. only (n = 5/82)	Neither COVID-19 inf. nor vac. (n = 2/82)
Number of patients (*n*)	82	47	28	5	2
Female, % (counts/*n*)	53.7 (44/82)	40.4 (19/45)	75.0 (21/28)	60.0 (3/5)	50.0 (1/2)
Age (years) median (25–75% percentiles)	52 (39.5–62.8)	57 (48.5–69.0)	43 (32.8–54.0)	52 (39.0–53.0)	26 (26.0–26.0)
BMI (kg/m^^2^^) median (25–75% percentiles)	26.3 (24.1–30.1)	26.2 (24.7–29.3)	27.3 (21.9–30.6)	25.5 (24.2–28)	26.6 (23.9–29.3)
**Cardiovascular risk factors** % (counts/*n*)	74.4 (61/82)	85.1 (40/47)	60.7 (17/28)	80.0 (4/5)	0 (0/2)
None % (counts/*n*)	25.6 (21/82)	14.9 (7/47)	39.3 (11/28)	20.0 (1/5)	100 (2/2)
Hypertension % (counts/*n*)	53.7 (44/82)	70.2 (33/47)	32.1 (9/28)	40.0 (2/5)	0 (0/2)
Diabetes % (counts/*n*)	8.5 (7/82)	10.6 (5/47)	7.1 (2/28)	0 (0/5)	0 (0/2)
Smoking % (counts/*n*)	8.5 (7/82)	10.6 (5/47)	7.1 (2/28)	0 (0/5)	0 (0/2)
Hypercholesterolaemia % (counts/*n*)	48.8 (40/82)	59.6 (28/47)	35.7 (10/28)	20.0 (2/5)	0 (0/2)
elevated Lipoprotein a % (counts/*n*)	11.0 (9/82)	10.6 (5/47)	14.3 (4/28)	0 (0/5)	0 (0/2)
**Atrial fibrillation** % (counts/*n*)	6.1 (5/82)	8.5 (4/47)	3.6 (1/28)	0 (0/5)	0 (0/2)
**Primary APS** % (counts/*n*)	76.8 (63/82)	85.1 (40/47)	67.9 (19/28)	20.0 (2/5)	100 (2/2)
Female % (counts/*n*)	50.8 (32/63)	40.0 (16/40)	73.7 (14/19)	50.0 (1/2)	50.0 (1/2)
**Secondary APS** % (counts/*n*)	23.2 (19/82)	14.9 (7/47)	32.1 (9/28)	60.0 (3/5)	0 (0/2)
Female % (counts/*n*)	63.2 (12/19)	42.9 (3/7)	77.8 (7/9)	66.7 (2/3)	0 (0/0)
Autoimmune disease ** % (counts/*n*)	35.4 (29/82)	25.5 (12/47)	46.4 (13/28)	60.0 (3/5)	50.0 (1/2)
Female % (counts/*n*)	69.0 (20/29)	50.0 (6/12)	84.6 (11/13)	66.7 (2/3)	100 (1/1)
**SLE** % (counts/*n*)	13.4 (11/82)	6.4 (3/47)	17.9 (5/28)	60.0 (3/5)	0 (0/2)
Female % (counts/*n*)	72.7 (8/11)	33.3 (1/3)	100.0 (5/5)	66.7 (2/3)	0 (0/0)
**Immunosuppressive drugs**	20.7 (17/82)	12.8 (6/47)	28.6 (8/28)	60.0 (3/5)	0 (0/2)
**VTE in medical history** % (counts/*n*)	79.3 (65/82)	68.1 (32/47)	92.8 (26/28)	100 (5/5)	100 (2/2)
**ATE in medical history** % (counts/*n*)	39.0 (32/82)	51.1 (24/47)	21.4 (6/28)	20.0 (2/5)	0 (0/2)
**Antiphospholipid antibodies**					
**Lupus anticoagulants** % (counts/*n*)	58.5 (48/82)	57.4 (27/47)	53.6 (15/28)	80.0 (4/5)	100 (2/2)
Elevated Mixcon LA Ratio % (counts/*n*)	53.7 (44/82)	51.1 (24/47)	50.0 (14/28)	80.0 (4/5)	100 (2/2)
Elevated DRVVT Ratio % (counts/*n*)	42.7 (35/82)	44.7 (21/47)	32.1 (9/28)	80.0 (4/5)	50 (1/2)
**Elevated aCL** % (counts/*n*)	80.5 (66/82)	80.9 (38/47)	82.1 (23/28)	60.0 (3/5)	100 (2/2)
Elevated aCL IgM % (counts/*n*)	43.9 (36/82)	48.9 (23/47)	39.3 (11/28)	20.0 (1/5)	50 (1/2)
Elevated aCL IgG % (counts/*n*)	54.9 (45/82)	51.1 (24/47)	57.1 (16/28)	60.0 (3/5)	100 (2/2)
**Elevated anti-2-GPI** % (counts/*n*)	69.5 (57/82)	70.2 (33/47)	71.4 (20/28)	60.0 (3/5)	50 (1/2)
Elevated anti-2-GPI IgM % (counts/*n*)	41.5 (34/82)	46.8 (22/47)	35.7 (10/28)	20.0 (1/5)	50 (1/2)
Elevated anti-2-GPI IgG % (counts/*n*)	46.3 (38/82)	44.7 (21/47)	46.4 (13/28)	60.0 (3/5)	50 (1/2)
**Elevated anti-2-GPI domain I IgG** % (counts/*n*)	34.2 (28/82)	25.5 (12/47)	42.9 (12/28)	60.0 (3/5)	50 (1/2)
**aPL status**					
Single-positive % (counts/*n*)	37.8 (31/82)	38.3 (18/47)	35.7 (10/28)	20.0 (2/5)	50 (1/2)
Double-positive % (counts/*n*)	23.2 (19/82)	23.4 (11/47)	25.0 (7/28)	20.0 (1/5)	0 (0/2)
Triple-positive % (counts/*n*)	39.0 (32/82)	38.3 (18/47)	39.3 (11/28)	20.0 (2/5)	50 (1/2)

* Twenty-six patients were vaccinated first and had their COVID-19 infection after at least two vaccinations whereas two patients had their COVID-19 infection prior to vaccination; ** Autoimmune diseases associated with APS are SLE (n = 11), mixed connective tissue disease (n = 7) or Sjögren’s syndrome (n = 1), autoimmune diseases not associated with APS are idiopathic thrombocytopenic purpura (n = 4), hemolytic anemia (n = 1), rheumatoid arthritis (n = 1), multiple sclerosis (n = 1), ankylosing spondylitis (n = 1), Hashimoto’s thyroiditis (n = 2), and autoimmune heparin-induced thrombocytopenia (n = 1), multiple diseases possible; BMI: body mass index; APS: antiphospholipid syndrome; SLE: systemic lupus erythematodes; VTE: venous thromboembolism; ATE: arterial thromboembolism; DRVVT: Dilute Russell’s viper venom time; aCL: anticardiolipin antibody; anti-β2-GPI: anti-β-2-glycoprotein I antibody; aPL: antiphospholipid antibody; vac.: vaccination; inf.: infection.

**Table 2:** Anticoagulant and/or antiplatelet medication in the total study population (n = 82) before (baseline) and after COVID-19 vaccination and/or infection (follow-up).

Medication	Baseline	Follow-Up
VKA only % (counts/*n*)	34.1 (28/82)	34.1 (28/82)
DOAC only % (counts/*n*)	32.9 (27/82)	32.9 (27/82)
LMWH only % (counts/*n*)	8.5 (7/82)	6.1 (5/82)
Fondaparinux only % (counts/*n*)	1.2 (1/82)	2.4 (2/82)
PI only % (counts/*n*)	7.3 (6/82)	7.3 (6/82)
VKA + PI % (counts/*n*)	7.3 (6/82)	7.3 (6/82)
DOAC + PI % (counts/*n*)	4.9 (4/82)	6.1 (5/82)
LMWH + PI % (counts/*n*)	2.4 (2/82)	1.2 (1/82)
none	1.2 (1/82)	2.4 (2/82)

VKA: Vitamin K antagonists (phenprocoumon, warfarin); DOAC: direct oral anticoagulants (rivaroxaban, apixaban); LMWH: low molecular weight heparin (enoxaparin, tinzaparin, dalteparin); PI: platelet inhibitors (acetylsalicylic acid, ticagrelor, prasugrel, clopidogrel).

**Table 3:** The patients’ characteristics after COVID-19 vaccination and/or infection (follow-up).

	Total Study Population (n = 82)	COVID-19 vac. only (n = 47/82)	COVID-19 vac. and inf. ** (n = 28/82)	COVID-19 inf. only (n = 5/82)	Neither COVID-19 inf. nor vac. (n = 2/82)
Time between baseline and Follow-up blood sampling (days) median (25–75% percentiles)	617 (486.5–783)	624 (518.5–814.5)	634 (494.8–747.8)	329 (253–334)	306 (241.3–369.8)
Time between baseline blood sampling and first COVID-19 vac. or inf. (days) median (25–75% percentiles)	200 (102.5–354.0)	218 (116–402)	194 (55.8–351.5)	93 (72.3–113.5)	305.5 ^###^ (n.a.)
Time between last COVID-19 vac. or inf. and Follow-up blood sampling (days) median (25–75% percentiles)	142 (88.5–199)	155 (114–208.5)	120 (56.3–159.8)	169 (155.3–205.5)	305.5 ^###^ (n.a.)
**≥1 COVID-19 vac.** % (counts/*n*)	91.5 (75/82)	100 (47/47)	100 (28/28)	n.a.	n.a.
Hospitalization after COVID-19 vac. % (counts/*n*)	5.3 (4/75)	8.5 (4/47)	0 (0/28)	n.a.	n.a.
≥1 SARS-CoV-2 inf. * % (counts/*n*)	40.2 (33/82)	n.a.	100 (28/28)	100 (5/5)	n.a.
Hospitalization after COVID-19 inf. % (counts/*n*)	9.1 (3/33)	n.a.	7.1 (2/28)	20 (1/5)	n.a.
of whom % were not vaccinated (counts/*n*)	33.3 (1/3)	n.a.	n.a.	100 (1/1)	n.a.
Infection in 2020 % (counts/*n*)	2.9 (1/35 ^#^)	n.a.	3.3 (1/30 ^##^)	0 (0/5)	n.a.
Infection in 2021 % (counts/*n*)	22.9 (8/35 ^#^)	n.a.	20.0 (6/30 ^##^)	40 (2/5)	n.a.
Infection in 2022 % (counts/*n*)	74.3 (26/35 ^#^)	n.a.	76.7 (23/30 ^##^)	60 (3/5)	n.a.
COVID-19 inf. on immunosuppressive drugs % (counts/*n*)	27.3 (9/33)	n.a.	21.4 (6/28)	60 (3/5)	n.a.
**Immunosuppressive drugs**	18.3 (15/82)	12.8 (6/47)	21.4 (6/28)	60.0 (3/5)	0 (0/2)
of whom % (counts/*n*) were infected with COVID-19	60.0 (9/15)	n.a.	100 (6/6)	100 (3/3)	n.a.
**VTE during observation** % (counts/*n*)	0.0 (0/82)	0.0 (0/47)	0.0 (0/28)	0 (0/5)	0 (0/2)
**ATE during observation** % (counts/*n*)	1.2 (1/82)	2.1 (1/47)	0.0 (0/28)	0 (0/5)	0 (0/2)
**Major bleeding during observation** % (counts/*n*)	1.2 (1/82)	2.1 (1/47)	0.0 (0/28)	0 (0/5)	0 (0/2)
**Antiphospholipid antibodies**					
**Lupus anticoagulants** % (counts/*n*)	61 (50/82)	57.4 (27/47)	60.7 (17/28)	80 (4/5)	100 (2/2)
Elevated Mixcon LA Ratio % (counts/*n*)	57.3 (47/82)	53.2 (25/47)	57.1 (16/28)	80 (4/5)	100 (2/2)
Elevated DRVVT Ratio % (counts/*n*)	42.7 (35/82)	38.3 (18/47)	46.4 (13/28)	60 (3/5)	50 (1/2)
**Elevated aCL** % (counts/*n*)	81.7 (67/82)	80.9 (38/47)	89.3 (25/28)	60 (3/5)	50 (1/2)
Elevated aCL IgM % (counts/*n*)	42.7 (35/82)	48.9 23/47)	39.3 (11/28)	0 (0/5)	50 (1/2)
Elevated aCL IgG %(counts/*n*)	51.2 (42/82)	44.7 (21/47)	60.7 (17/28)	60 (3/5)	50 (1/2)
**Elevated anti-2-GPI** % (counts/*n*)	63.4 (52/82)	61.7 (29/47)	67.9 (19/28)	60 (3/5)	50 (1/2)
Elevated anti-2-GPI IgM % (counts/*n*)	40.2 (33/82)	44.7 (21/47)	39.3 (11/28)	0 (0/5)	50 (1/2)
Elevated anti-2-GPI IgG % (counts/*n*)	41.5 (34/82)	40.4 (19/47)	39.3 (11/28)	60 (3/5)	50 (1/2)
**Elevated anti-2-GPI domain I IgG** % (counts/*n*)	37.8 (31/82)	29.8 (14/47)	50.0 (14/28)	40 (2/5)	50 (1/2)
**aPL status**					
Single-positive % (counts/*n*)	36.6 (30/82)	38.3 (18/47)	32.1 (9/28)	40 (2/5)	50 (1/2)
Double-positive % (counts/*n*)	18.3 (15/82)	17.0 (8/47)	21.4 (6/28)	20 (1/5)	0 (0/2)
Triple-positive % (counts/*n*)	40.2 (33/82)	38.3 (18/47)	42.9 (12/28)	40 (2/5)	50 (1/2)
**Loss of aPL** % (counts/*n*)	4.9 (4/82)	6.4 (3/47)	3.6 (1/28)	0 (0/5)	0 (0/2)
**Increase ^+^ in aPL positivity** % (counts/*n*)	7.3 (6/82)	4.3 (2/47)	14.3 (4/28)	0 (0/5)	0 (0/2)
**Decrease ^++^ in aPL positivity** % (counts/*n*)	14.6 (12/82)	17.0 (8/47)	14.3 (4/28)	0 (0/5)	0 (0/2)

* PCR or rapid antigen test positive; ** Twenty-six patients were vaccinated first and had their COVID-19 infection after at least two vaccinations whereas two patients had their COVID-19 infection prior to vaccination; ^#^ Number of total infections summed up to 35 because two patients were infected twice; ^##^ Number of total infections summed up to 30 because two patients were infected twice; ^###^ Interval (days) between the baseline and follow-up blood testing; ^+^ Increase in aPL positivity: increase in the amount of aPL antibody-class positivity; ^++^ Decrease in aPL positivity: decrease in the amount of aPL antibody-class positivity; n.a.: not applicable; vac.: vaccination; inf.: infection; VTE: venous thromboembolism; ATE: arterial thromboembolism; DRVVT: Dilute Russell’s viper venom time; aCL: anticardiolipin antibody; anti-β2-GPI: anti-beta-2-glycoprotein I antibody; aPL: antiphospholipid antibody.

**Table 4:** Effect of COVID-19 vaccination and/or infection on antiphospholipid antibodies and platelet count. Comparisons of different parameters at baseline and follow-up.

	Total Study Population			COVID-19 vac. only			COVID-19 vac. and inf. **			COVID-19 inf. only		
	Median (25–75% Percentiles)			Median (25–75% Percentiles)			Median (25–75% Percentiles)			Median (25–75% Percentiles)		
	Baseline	Follow-Up	*p*	Baseline	Follow-Up	*p*	Baseline	Follow-Up	*p*	Baseline	Follow-Up	*p*
**Lupus anticoagulants**												
DRVVT Ratio VKA+ * (Normal range: 0.9–1.6)	1.9 (1.4–2.0)	1.6 (1.4–2.1)	0.494	1.5 (1.3–2.0)	1.6 (1.4–1.8)	0.313	1.9 (1.4–2.0)	1.8 (1.4–2.4)	0.529	1.9 (1.9–2.0)	1.9 (1.7–2.1)	1.0
DRVVT Ratio VKA- * (Normal range: 0.9–1.2)	1.6 (1.3–1.9)	1.6 (1.3–1.7)	0.135	1.5 (1.3–1.8)	1.5 (1.3–1.6)	0.339	1.8 (1.5–2.4)	1.7 (1.4–2.3)	0.584	n.a.	n.a.	n.a.
Mixcon LA Ratio (Normal range 0.8-1.07)	1.3 (1.2–1.7)	1.3 (1.2–1.8)	0.398	1.3 (1.2–1.7)	1.3 (1.3–1.6)	0.319	1.3 (1.1–1.6)	1.3 (1.1–1.6)	0.127	2.7 (2.3–2.9)	2.0 (1.8–2.1)	0.181
**anti-2-GPI**												
anti-ß2-GPI domain I IgG (U/mL) (Normal range: 0–20)	5.6 (3.6–55.4)	6.3 (3.6–61.5)	0.858	3.8 (3.6–22.2)	3.6 (3.6–24.7)	1.0	12.2 (3.7–91.2)	16.9 (3.6–99.1)	0.723	49.0 (3.6–121.0)	6.0 (3.6–152.2)	0.423
anti-2-GPI IgM (U/mL) (Normal range 0–12)	5.5 (1.5–22.5)	5.5 (1.4–33.3)	0.129	8.0 (2.2–26.6)	9.1 (2.3–28.4)	0.550	3.6 (1.4–19.5)	3.1 (1.3–47.4)	0.033	5.0 (1.5–5.4)	5.3 (1.4–6.2)	1.0
anti-2-GPI IgG (U/mL) (Normal range 0–17.4)	14.6 (8.6–167.3)	12.5 (6.6–249.3)	0.021	14.9 (9.7–66.3)	12.0 (7.4–45.0)	0.031	18.3 (8.7–558.3)	12.5 (6.9–424.4)	0.537	47.1 (3.7–665.7)	41.9 (4.0–451.4)	0.438
**aCL**												
aCL IgM (U/mL) (Normal range: 0–18.7)	10.8 (4.2–43.8)	8.1 (3.5–51.1)	0.901	14.8 (4.4–55.0)	14.3 (4.1–56.9)	0.645	7.8 (2.6–30.3)	7.6 (2.7–48.7)	0.039	5.6 (4.9–7.7)	4.3 (4.2–6.7)	0.188
aCL IgG (U/mL) (Normal range: 0–13.6)	17.3 (5.1–72.4)	15.8 (4.0–53.7)	<10^−3^	14.8 (4.6–48.0)	11.1 (2.7–39.0)	<10^−3^	32.8 (5.2–133.3)	29.5 (7.9–106.8)	0.284	44.4 (13.2–113.7)	24.7 (13.2–101.0)	0.100
**Platelet count (10^3^/µL)** (Normal range 150–400)	212.5 (174.0–249.0)	221.5 (176.8–267.3)	0.608	203.5 (174.8–242.0)	207.0 (170.3–254.8)	0.645	221.0 (177.5–258.5)	236.0 (209.5–283.5)	0.546	231.0 (145.0–243.0)	237.0 (182.0–257.0)	0.313

* DRVVT ratio reference values differed in patients with or without vitamin K antagonist (VKA) treatment. VKA+: patients on VKA treatment; VKA−: patients without VKA treatment; ** Twenty-six patients were vaccinated first and had their COVID-19 infection after at least two vaccinations whereas two patients had their COVID-19 infection prior to vaccination; anti-β2-GPI: anti-β2-glycoprotein I antibody; aCL: anticardiolipin antibody; inf.: infection; vac.: vaccination; n.a.: not applicable due to missing data.

**Table 5:** Patients being hospitalized due to COVID-19 vaccination or COVID 19-infection with a distinction between mild and severe cases.

Patient	Age (Years)	Sex	Hospitalization after vac. or inf.	Time from vac. or inf. to Hospitalization (Days)	Diagnosis/Symptoms Leading to Hospitalization	Duration of Hospitalization (Days)	Outcome
**Mild Cases**							
1	77	female	inf. *	12	nausea, vomiting, headache	5	fully recovered
2	38	female	inf. *	11	esophagitis	1	fully recovered
3	45	female	vac.	37	spinning vertigo, periods of aphasia, vomiting	3	fully recovered
4	71	male	vac.	2	acute hearing loss	n.a.	hearing 75% restored after 3 weeks
**Severe cases**							
5	60	female	inf. **	1	acute general distress, later respiratory insufficiency	51	stable but impaired general condition
6	85	male	vac.	5	arm paresis left side due to paramedian pons infarction	19	stable but impaired general condition
7	54	male	vac.	33	dysaesthesia right hand and aphasia due to spontaneous subdural hematoma	19	symptoms have improved, not fully recovered

inf.: COVID-19 infection; vac.: COVID-19 vaccination; n.a.: Not applicable due to missing data; * patient had been vaccinated with Comirnaty^®^ by BioNTech/Pfizer at least twice before; ** The patient had not received any vaccination prior to COVID-19 infection.

## Data Availability

Not applicable.

## References

[1] Linnemann B. (2018). Antiphospholipid syndrome-an update. Vasa.

[2] Miyakis S., Lockshin M.D., Atsumi T., Branch D.W., Brey R.L., Cervera R., Derksen R.H., PG D.E.G., Koike T., Meroni P.L. (2006). International consensus statement on an update of the classification criteria for definite antiphospholipid syndrome (APS). J. Thromb. Haemost..

[3] Wang X., Gkrouzman E., Andrade D.C.O., Andreoli L., Barbhaiya M., Belmont H.M., Branch D.W., de Jesus G.R., Efthymiou M., Rios-Garces R. (2021). COVID-19 and antiphospholipid antibodies: A position statement and management guidance from AntiPhospholipid Syndrome Alliance for Clinical Trials and InternatiOnal Networking (APS ACTION). Lupus.

[4] Paul-Ehrlich-Institut COVID-19-Impfstoffe. https://www.pei.de/DE/arzneimittel/impfstoffe/covid-19/covid-19-node.html.

[5] Gendron N., Dragon-Durey M.A., Chocron R., Darnige L., Jourdi G., Philippe A., Chenevier-Gobeaux C., Hadjadj J., Duchemin J., Khider L. (2021). Lupus Anticoagulant Single Positivity During the Acute Phase of COVID-19 Is Not Associated With Venous Thromboembolism or In-Hospital Mortality. Arthritis Rheumatol..

[6] Uthman I.W., Gharavi A.E. (2002). Viral infections and antiphospholipid antibodies. Semin Arthritis Rheum.

[7] Devreese K.M.J., Linskens E.A., Benoit D., Peperstraete H. (2020). Antiphospholipid antibodies in patients with COVID-19: A relevant observation?. J. Thromb. Haemost..

[8] Galeano-Valle F., Oblitas C.M., Ferreiro-Mazon M.M., Alonso-Munoz J., Del Toro-Cervera J., di Natale M., Demelo-Rodriguez P. (2020). Antiphospholipid antibodies are not elevated in patients with severe COVID-19 pneumonia and venous thromboembolism. Thromb. Res..

[9] Borghi M.O., Beltagy A., Garrafa E., Curreli D., Cecchini G., Bodio C., Grossi C., Blengino S., Tincani A., Franceschini F. (2020). Anti-Phospholipid Antibodies in COVID-19 Are Different From Those Detectable in the Anti-Phospholipid Syndrome. Front. Immunol..

[10] Espinosa G., Zamora-Martinez C., Perez-Isidro A., Neto D., Bravo-Gallego L.Y., Prieto-Gonzalez S., Vinas O., Moreno-Castano A.B., Ruiz-Ortiz E., Cervera R. (2022). Persistent Antiphospholipid Antibodies Are Not Associated With Worse Clinical Outcomes in a Prospective Cohort of Hospitalised Patients With SARS-CoV-2 Infection. Front. Immunol..

[11] Zuo Y., Estes S.K., Ali R.A., Gandhi A.A., Yalavarthi S., Shi H., Sule G., Gockman K., Madison J.A., Zuo M. (2020). Prothrombotic autoantibodies in serum from patients hospitalized with COVID-19. Sci. Transl. Med..

[12] Gan G., Liu H., Liang Z., Zhang G., Liu X., Ma L. (2022). Vaccine-associated thrombocytopenia. Thromb. Res..

[13] Iba T., Levy J.H. (2022). Thrombosis and thrombocytopenia in COVID-19 and after COVID-19 vaccination. Trends Cardiovasc. Med..

[14] Pontara E., Banzato A., Bison E., Cattini M.G., Baroni G., Denas G., Calligaro A., Marson P., Tison T., Ruffatti A. (2018). Thrombocytopenia in high-risk patients with antiphospholipid syndrome. J. Thromb. Haemost..

[15] Schaefers C., Paulsen F.O., Frenzel C., Weisel K., Bokemeyer C., Seidel C. (2023). Increased incidence of immune thrombocytopenia (ITP) in 2021 correlating with the ongoing vaccination campaign against COVID-19 in a tertiary center-A monocentric analysis. Br. J. Haematol..

[16] Lippi G., Plebani M., Henry B.M. (2020). Thrombocytopenia is associated with severe coronavirus disease 2019 (COVID-19) infections: A meta-analysis. Clin. Chim. Acta.

[17] Yang M., Ng M.H., Li C.K. (2005). Thrombocytopenia in patients with severe acute respiratory syndrome (review). Hematology.

[18] Franchini M., Veneri D., Lippi G. (2017). Thrombocytopenia and infections. Expert Rev. Hematol..

[19] Chen Y., Xu Z., Wang P., Li X.M., Shuai Z.W., Ye D.Q., Pan H.F. (2022). New-onset autoimmune phenomena post-COVID-19 vaccination. Immunology.

[20] Yazdanpanah N., Rezaei N. (2022). Autoimmune complications of COVID-19. J. Med. Virol..

[21] Pascolini S., Vannini A., Deleonardi G., Ciordinik M., Sensoli A., Carletti I., Veronesi L., Ricci C., Pronesti A., Mazzanti L. (2021). COVID-19 and Immunological Dysregulation: Can Autoantibodies be Useful?. Clin. Transl. Sci..

[22] Gomez-Puerta J.A., Martin H., Amigo M.C., Aguirre M.A., Camps M.T., Cuadrado M.J., Hughes G.R.V., Khamashta M.A. (2005). Long-term follow-up in 128 patients with primary antiphospholipid syndrome: Do they develop lupus?. Medicine (Baltimore).

[23] Zou Y., Guo H., Zhang Y., Zhang Z., Liu Y., Wang J., Lu H., Qian Z. (2020). Analysis of coagulation parameters in patients with COVID-19 in Shanghai, China. Biosci. Trends.

[24] Fiolet T., Kherabi Y., MacDonald C.J., Ghosn J., Peiffer-Smadja N. (2022). Comparing COVID-19 vaccines for their characteristics, efficacy and effectiveness against SARS-CoV-2 and variants of concern: A narrative review. Clin. Microbiol. Infect..

[25] Agency U.H.S. COVID-19 Vaccine Surveillance Report Week 44. https://assets.publishing.service.gov.uk/government/uploads/system/uploads/attachment_data/file/1115385/Vaccine_surveillance_report___week-44.pdf.

[26] Statista Verteilung Besorgniserregender Coronavirusvarianten (VOC) in Deutschland 2021. https://de.statista.com/statistik/daten/studie/1208627/umfrage/ausbreitung-von-corona-mutationen-in-deutschland/.

[27] Robert Koch Institut SARS-CoV-2: Virologische Basisdaten Sowie Virusvarianten. https://www.rki.de/DE/Content/InfAZ/N/Neuartiges_Coronavirus/Virologische_Basisdaten.html;jsessionid=CBB1C1C394B7CDCE980B01F79385A939.internet062?nn=13490888#doc14716546bodyText8.

[28] Greinacher A., Thiele T., Warkentin T.E., Weisser K., Kyrle P.A., Eichinger S. (2021). Thrombotic Thrombocytopenia after ChAdOx1 nCov-19 Vaccination. N. Engl. J. Med..

[29] Carli G., Nichele I., Ruggeri M., Barra S., Tosetto A. (2021). Deep vein thrombosis (DVT) occurring shortly after the second dose of mRNA SARS-CoV-2 vaccine. Intern. Emerg. Med..

[30] Schulz A., Herrmann E., Ott O., Lindhoff-Last E. (2022). Thromboembolic Antiphospholipid Syndrome (APS): Efficacy and Safety of Different Anticoagulants-Results of the APSantiCO Registry. J. Clin. Med..

[31] Institut R.K. Gesamtübersicht der pro Tag ans RKI Übermittelten Fälle und Todesfälle. https://www.rki.de/DE/Content/InfAZ/N/Neuartiges_Coronavirus/Daten/Fallzahlen_Gesamtuebersicht.html.

[32] Kaatz S., Ahmad D., Spyropoulos A.C., Schulman S., For the Subcommittee on Control of Anticoagulation (2015). Definition of clinically relevant non-major bleeding in studies of anticoagulants in atrial fibrillation and venous thromboembolic disease in non-surgical patients: Communication from the SSC of the ISTH. J. Thromb. Haemost..

